# Cancer-testis antigens, semenogelins 1 and 2, exhibit different anti-proliferative effects on human lung adenocarcinoma cells

**DOI:** 10.1038/s41420-020-00336-5

**Published:** 2020-10-22

**Authors:** Oleg Shuvalov, Alyona Kizenko, Alexey Petukhov, Nickolai Aksenov, Olga Fedorova, Mikhail Vorobev, Alexandra Daks, Nickolai Barlev

**Affiliations:** 1 Institute of cytology RAS, St-Petersburg, Russia; 2 Almazov National Medical Research Centre, St-Petersburg, Russia; 3 MIPT, Doloprudny, Moscow region, Russia

**Keywords:** Mechanisms of disease, Oncogenes

## Abstract

Сancer-testis antigens (CTAs) comprise proteins which are aberrantly expressed in various malignancies, yet under normal situation are restricted to only germ cells. Semenogelins 1 and 2 (SEMG1 and 2, respectively) belong to the family of non-X-linked (autosomal) cancer-testis antigens. They are the major protein ingredients of human semen and share 78% of similarity between them on the gene level. SEMG1/2 gene products regulate the motility and fertility of sperm, as well as provide sperm the antibacterial defense. Besides, SEMG1 and SEMG2 were detected in various malignancies including small cell lung cancer (SCLC). However, the biological role of both SEMG1 and 2 proteins in tumorigenesis has not been fully understood. We demonstrate here that SEMG1 and SEMG2 (SEMGs) exhibit different patterns of expression and sub-cellular localization in non-small cell lung cancer (NSCLC) cell lines. To elucidate the biological properties of SEMGs in NSCLC, we established H1299 cell lines that were stably transduced with either SEMGs-overexpressing or knockdown vectors, respectively. Using fluorescence-based dihydroethidium (DHE) assay we showed that both SEMGs augmented the production of reactive oxygen species (ROS) up to 2 times. Moreover, SEMGs (especially SEMG1) strongly increased the number of Annexin V–positive apoptotic cells manifesting an increased sensitivity to genotoxic drugs including doxorubicin, etoposide, and cisplatin. Taken our results together, SEMGs may arguably play a positive role in tumorigenesis by sensitizing NSCLCs to genotoxic therapy.

## Introduction

Semenogelins 1 and 2 are referred to Cancer-Testis Antigens (CTAs) and represent a group of proteins which are frequently expressed in various neoplasms but normally are restricted to germ cells^1^.

CTAs are frequently associated with aggressive tumors at the late stage of their development^2–4^. Due to the immune privilege status of the testis tissue, the re-expression of CTAs in tumors often induces strong immune response. This makes CTAs perspective candidates for immunotherapy^5^.

On the one hand, expression of CTAs in tumor cells is deemed as the consequence of global aberration in the gene expression program. In normal cells the expression of CTAs is silenced by methylation^6^ and is re-activated in cancer due to hypomethylation of the corresponding loci. Indeed, the major molecular mechanisms regulating CTAs expression are hypomethylation of DNA and histone post-translational modifications^5^. An observation that different CTAs are frequently expressed in the same tumors favors this hypothesis.

On the other hand, a number of evidences indicate that the expression of certain CTAs actively contributes to the development of tumors. Accordingly, CTAs were shown to down-regulate apoptosis^7,8^, induce proliferation^9,10^, and migration and invasion of cancer cells^11,12^.

Indeed, actively proliferating, migrating and invading cancer cells resemble germ cells that also possess these properties. In addition, certain tumor cells, especially low-grade ones, often express gene patterns similar to embryonic stem cells^13^.

Semenogelins 1 and 2 (SEMG1 and 2, respectively) are the most abundant proteins of human sperm. SEMG1 is 50-kDa and SEMG2 is 63 kDa proteins, respectively. They both are secreted to semen inside seminal vesicles and then are rapidly degraded by the prostate specific antigen (PSA, a.k.a. kallikrein peptidase) to small peptides. PSA is an androgen-dependent 30KDa glycoprotein that possesses chymotrypsin-like enzymatic activity and plays a major role in the fragmentation of semenogelins^14^. Unlike proteasomes that recognize a wide spectrum of ubiquitinated targets, PSA digests only a limited number of substrates, including TGF-beta and IGFBP^15,16^. In semenal fluid, semenogelins (SEMGs) and products of their proteolysis perform a number of important functions. They regulate the motility^17^ and fertility of sperm^18^, as well as provide sperm the antibacterial defense^19^.

Almost all information on the role of SEMGs in reproduction is devoted to SEMG1, whereas the biological activity of SEMG2 remains largely unknown despite the fact that they share 78% similarity between them. Regarding their involvement in tumorigenesis, the only information available is that SEMG1 is a co-activator of androgen receptor in prostate cancer^20^.

SEMGs have been detected in various malignancies^21–23^ including lung cancer. SEMGs are secreted outside from SCLC cells^24^ and can be detected in the serum of NSCLC patients^25^. Therefore, we decided to obtain insights into their role in cancer cells by examining the expression patterns of both SEMGs using a panel of NSCLC cell lines and by assessing effects of SEMGs on migration, survival, and resistance to genotoxic chemotherapeutics.

## Materials and methods

### Cell culture and manipulations

Human lung cancer cells lines (A549, H460, H1650, H522, H520 and H1299) were purchased from ATCC. They were cultivated in RPMI medium supplemented with 10% fetal bovine serum (FBS), 100 units/ml penicillin, 100 μg/ml streptomycin, and 2 mM l-glutamine. Cells were grown at 37 °C in 5% CO_2_ atmosphere.

### Plasmids and cloning

Full-length CDS sequences of both human SEMG1 (NM_003007.4) and SEMG2 (NM_003008.2) were amplified by PCR from cDNA derived from MCF7 cell lines with follows primers including restriction sites for subsequent cloning: SEMG1_forward 5′-ATTGAATTCATGAAGCCCAACATCATCTTTGTAC-3′, SEMG1_reverse 5′-ATTCTCGAGTGTAAATAATGGGTTTCGGTCGTTG-3′, SEMG2_forward ACCGCGGCCGCTAGATGAAGTCCATCATCCTCTTTGTCC, SEMG2_reverse TTCTCGAGTGTAGATATTGGATTTCTGTCTTCATTATATTGTTG. Amplified sequences were digested by *EcoR*I/*Xho*I (in the case of SEMG1) or *Not*I/*Xho*I (for SEMG2) and cloned to Pires-hr-1a vector in fusion with 3x-Flag tag. Then, sequences of 3xFlag-SEMG1 and 3xFlag-SEMG2 were cut by *EcoR*I/*Pml*I and *Not*I/*Pml*I and subcloned to LegoIG2 lentiviral vector (which allow selection by GFP fluorescence) purchased from Addgene and then were checked by sequencing.

To knockdown SEMG2, lentiviral pGreenPuro vector bearing sh hairpin (5′-GCAAGTCTCAAAACCAGGTAACAATTCAT-3′) or scramble (5′-CCTAAGGTTAAGTCGCCCTCG-3′) were used.

### Western-blotting

For western-blotting following antibodies were used: anti-SEMG1 (1:1000, PA5-30168, Invitrogen), anti-SEMG2 (1:1000, PA5-42099, Invitrogen), anti-Flag (1:1000, M2, Sigma, USA) and anti-β-actin (1:1000, A-2228, Sigma, USA). The secondary antibodies were anti-mouse and anti-rabbit (1:10,000; Sigma, USA).

### Immunofluorescence

Cells grown on glass cover slips were fixed with 4% PFA in PBS for 15 min and then washed three times in PBS and incubated for 60 min with permeabilization blocking solution (5% BSA, 0.3% Triton X-100 in PBS) at room temperature. Cells were stained with anti-SEMG1 or anti-SEMG2 antibodies in permeabilization blocking solution for 16 h at 4 °C, washed three times in PBS and incubated with the secondary antibody in permeabilization blocking solution (goat anti-rabbit, AlexaFluor488 or 546, Invitrogen) for 1 h at room temperature and washed three times in PBS. Slides were mounted using ProLong Gold Antifade Mountant with DAPI (P36931, Invitrogen). Images were analyzed by confocal microscope (Leica).

### Measurement of ROS production

Endogenous ROS (predominantly, superoxide) were quantified by using Muse® ROS assay kit (EMD Millipore, USA) in accordance with manufacture’s protocol followed by flow cytometry using the Guava Easy Cyte 8 instrument (EMD Millipore, USA).

For validation, NAC (5 mM, 4 h) was added followed by DHE staining with subsequent fluorescent microscopy.

### Proliferation assay

For the proliferation assay, cell lines of interest were seeded in triplicates to a confluence of 30%. After three days, the number of cells in each case were counted using an automated cell counter Countess® (Invitrogen, USA).

### MTT assay

10,000 of cells were seeded to each well of 96-well plate for overnight. On the next day, doxorubicin (Sigma) or cisplatin (Teva) were added at different concentration for 48 h; 6 wells per sample were used. Then 10 μl of 5 mg/ml Triazolyl Blue solution was added to each well for 4 h at 37 °С. After removing MTT containing medium, 150 μl isopropyl alcohol (supplemented with 40 mM HCl and 0,1% NP-40) was added to dissolve MTT-formazan salt. The absorbance at 570 nm and 630 nm was measured using BioRad iMark microplate reader (BioRad, USA).

### Cell cycle analysis

Cells were harvested, washed with PBS, and fixed in 70% ethanol for 1 h. Staining for DNA content was performed with 50 μg/ml propidium iodide (Invitrogen, USA) and 1 μg/ml RNase A (ThermoFischer) for 30 min. Flow cytometry was performed using the Coulter EPICS XL Flow Cytometer (Beckman Coulter, USA). Data were analyzed using Win MDI software version 2.8 (Scripps Research Institute, USA).

### Annexin V assay

Analysis of apoptosis on the indicated cell lines was performed by Annexin V/propidium iodide double staining followed by Flow cytometry (Guava Technologies, Millipore, USA). The cells 2 days after transfection were collected and subjected to analysis. A minimum of 5000 cells were then analyzed by FACScan with guavaSoft 3.1.1 software (Guava Technologies, Millipore, USA) for acquisition and analysis in three independent biological replicates.

### Statistical analysis

All data are demonstrated as mean or standard error of the mean (SEM) ± standard deviation (SD) of at least three replicates. Statistical significance was analyzed using Student’s *t*-test, *P* < 0.05 was considered significant and is denoted as *.

## Results

### Semenogelins are expressed in human lung cancer cell lines

**Fig. 1:**
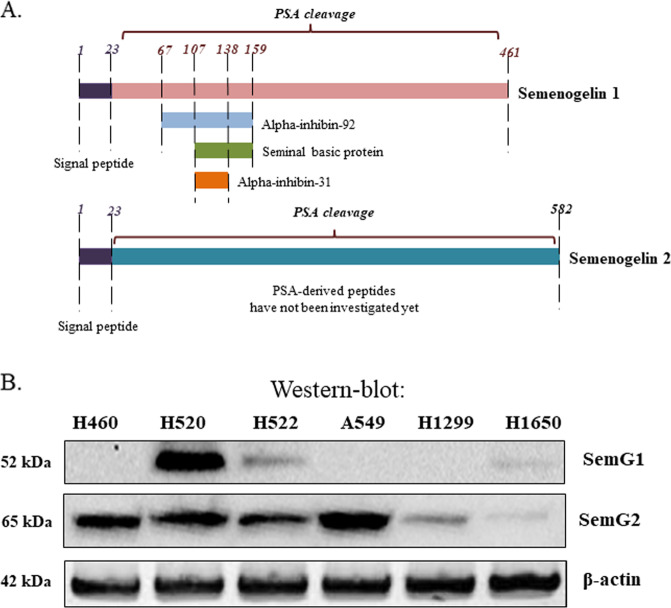
NSCLC cell lines express SEMG1 and SEMG2. **a** Comparison of SEMG1 and SEMG2. PSA – prostate specific antigen, cleaves SEMG1 and SEMG2 to small peptides indicated. **b** Distribution of SEMG1 and SEMG2 in different NSCLC cell lines. Western-blot analysis.

Earlier reports suggest that SEMGs are expressed in SCLC cell lines^24^. To expand our knowledge on SEMGs expression in NSCLCs on the protein level, we decided to check the presence of highly homologous SEMG1 and SEMG2 proteins (Fig. 1a) in the panel of 5 adenocarcinoma and 1 squamous cell carcinoma NSCLC cell lines.

As shown on Fig. 1b, SEMG1 was expressed only in H520, H522, and to some extend in H1650 cells, whereas SEMG2 was present in all cell lines tested. These data suggest that SEMGs are differentially expressed not only in SCLC but also in NSCLC.

### Semenogelins 1 and 2 have different subcellular localization depending on cell’s background

Semenogelins have been discovered in SCLC by analyzing the proteins recovered from the cell surface adhesion complexes^24^. However, the question of sub-cellular localization of SEMGs in cancer cells has never been addressed. Thus, we decided to examine the subcellular localization of SEMGs in our NSCLC cell lines.

**Fig. 2:**
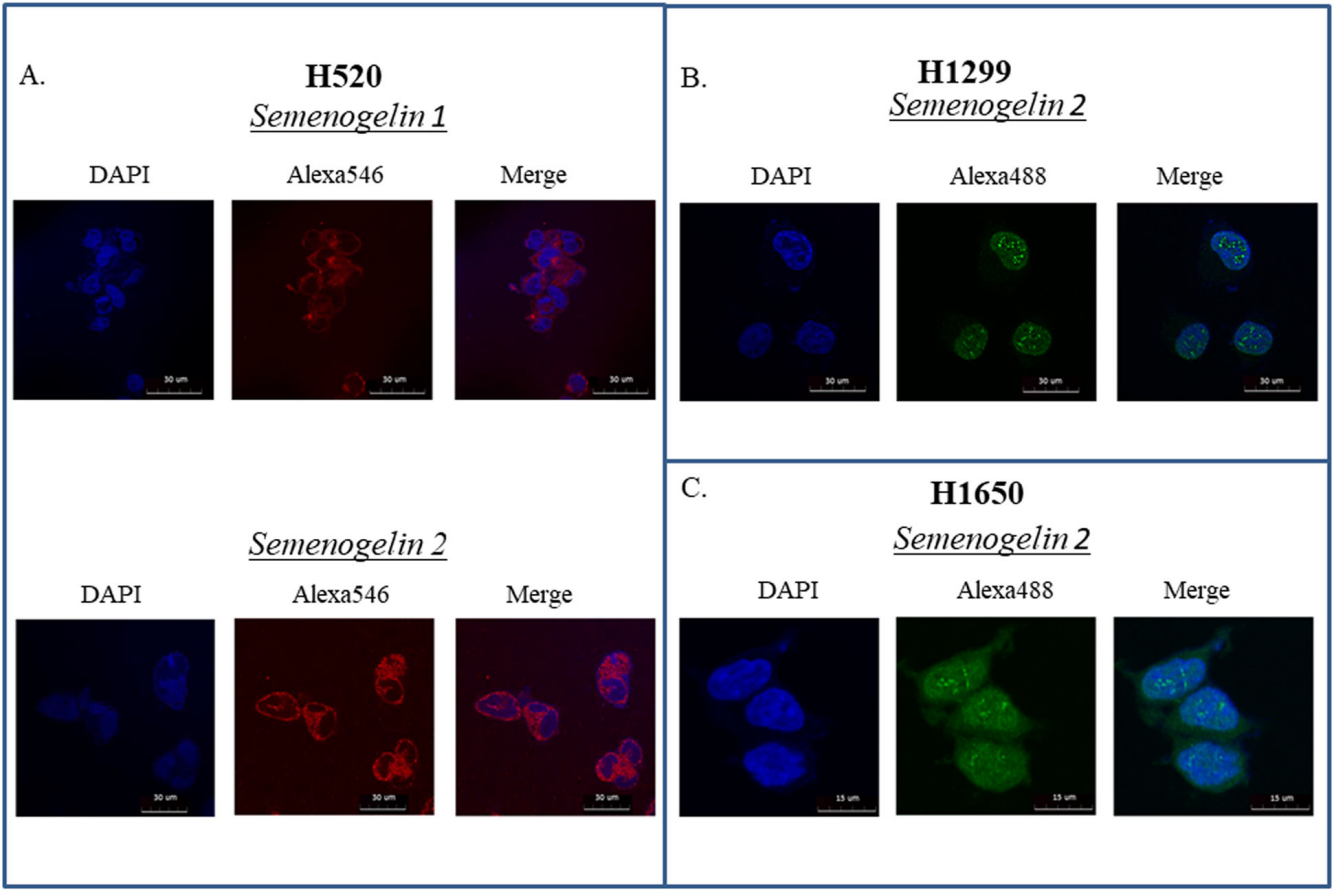
Immunofluorescence of SEMGs. **a** H520, **b** H1299 and **c** H1650 cells. Secondary antibodies conjugated with Alexa546 and Alexa 488 were used to visualize primary anti-SEMG1 and anti-SEMG2 antibodies.

We have carried out confocal immunofluorescence microscopy on H520, H1299 and H1650 cells using anti-SEMG1 and anti-SEMG2 antibodies. Figure 2a demonstrates that both SEMG1 and SEMG2 have cytoplasmic localization with uniform distribution in H520 squamous cell carcinoma cells. Interestingly, SEMG2 displayed nuclear dot-like localization in H1299 and H1650 adenocarcinoma cells (Fig. 2b, c). No SEMG1 staining was observed in H1299 and H1650 cells, which is in agreement with our western blot results.

Collectively, our results suggest that SEMG1 and SEMG2 have different sub-cellular localization depending on the cell context. Also, it is tempting to speculate that SEMG1 and SEMG2 may have different functions depending on the cell line.

### Overexpression of SEMGs inhibits proliferation of H1299 cells

**Fig. 3:**
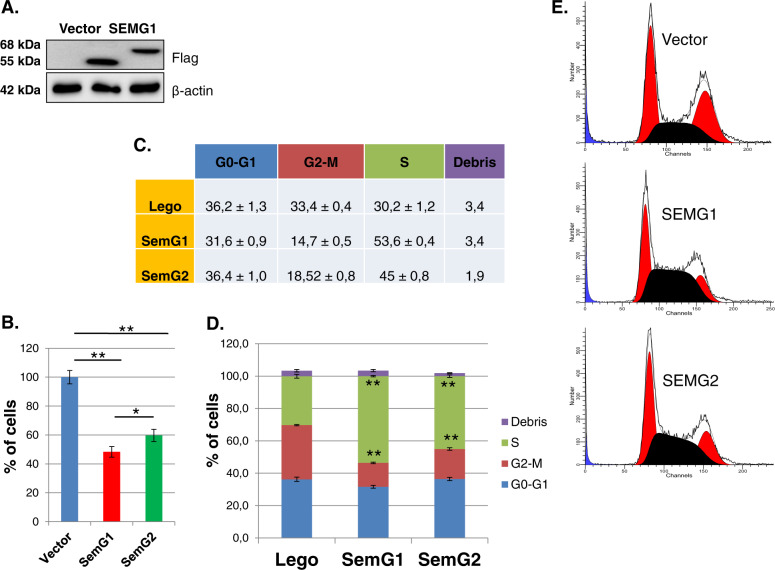
Overexpression of SEMG1 and SEMG2 inhibits proliferation and cell cycle progression of H1299 cells. **a** H1299 cells stably overexpressed SEMG1, SEMG2 or vehicle (western-blot). **b** Proliferation assay based on cell counting. Results are represented as mean ± SEM of five experiments. *P < 0.05; **P < 0.01; **c**–**e** Cell cycle analysis of H1299 cells overexpressing SEMG1, SEMG2 or vehicle. Results are represented as mean ± SEM of three experiments. *P < 0.05; **P < 0.01.

To investigate the biological effects of SEMGs in lung adenocarcinoma cell model, we have generated lentiviral-transduced H1299 cells with stable overexpression of SEMG1, SEMG2 or the corresponding vehicle as control (Fig. 3a).

Using these cell lines, we have demonstrated that overexpression of SEMG1 and SEMG2 inhibited proliferation of H1299 cells up to 52% and 40%, respectively (Fig. 3b). These data are also in concord with the results of cell cycle analysis. This analysis showed that both SEMGs significantly increased the number of cells in S-phase (Fig. 3c–e). Taken together, these results suggest that overexpression of SEMG1 and SEMG2 impedes the cell cycle progression of H1299 cells and inhibits their proliferation.

### Semenogelins up-regulate ROS production

It has been demonstrated previously^18^ that SEMGs possess ROS-scavenging activity during fertilization of sperm. Based on this, we decided to assess whether SEMGs have any impact on ROS utilization in the NSCLC cell model. To this end, we have used Muse® ROS detection kit, which allows quantification of ROS production (predominantly, superoxide) based on the DHE fluorescence.

**Fig. 4:**
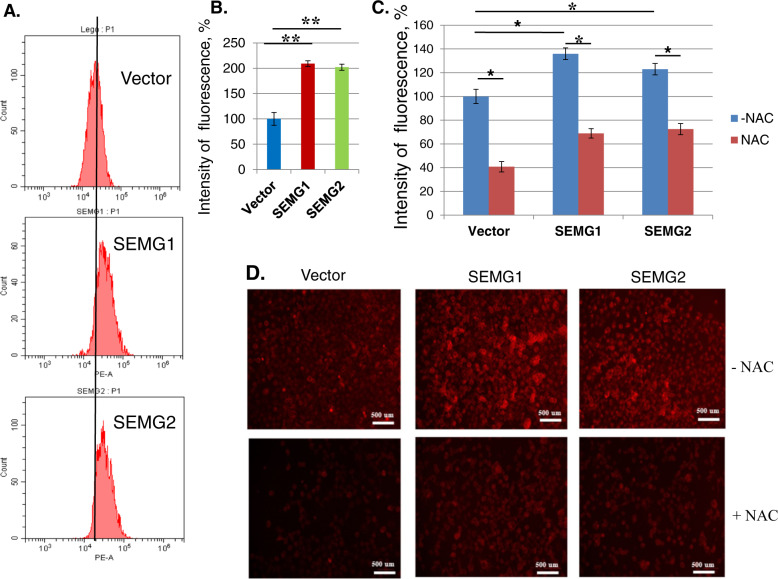
Overexpression of SEMG1 and SEMG2 up-regulates production of ROS. Cells were incubated with DHE and then analyzed by flow cytometry (**a**, **b**). Cells were pre-incubated or not with NAC, than were stained with DHE and analyzed by fluorescent microscopy (**c**, **d**) Results are represented as mean ± SEM of three experiments. **P < 0.01.

In contrast to our expectations, both SEMGs significantly enhanced DHE fluorescence (209% for SEMG1 and 202% for SEMG2 over control cells) (Fig. 4a, b) which, in turn, was compromised by treatment with NAC (Fig. 4c, d). These results suggest that overexpression of SEMG1 and SEMG2 up-regulates the production of ROS in H1299 cells.

### Semenogelins induce apoptosis and sensitize H1299 cells to genotoxic drugs

Exposure of cancer cells to genotoxic drugs often results in generation of ROS and increased oxidative stress^26^. Given our results that SEMGs overexpression activates ROS production, we were interested whether SEMGs also affect susceptibility of cancer cells to genotoxic drugs.

**Fig. 5:**
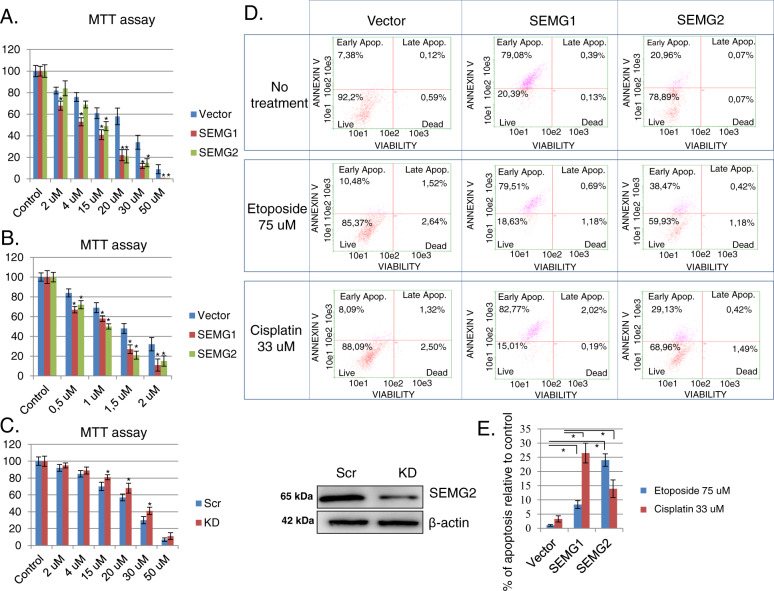
SEMGs induce apoptosis and sensitize cells to genotoxic drugs. MTT assay of H1299 cells overexpressing vector, SEMG1, SEMG2 and incubated with different concentrations of **a** doxorubicin or **b** cisplatin. **c** MTT assay of H1299 cells with scramble or SEMG2 knockdown (KD) treated with cisplatin. Knockdown of SEMG2 is visualized by western-blotting. **d** Apoptosis profile of non-treated or treated with 30 μM of cisplatin or 75 μM of etoposide H1299 cells overexpressing vector, SEMG1 or SEMG2. **e** Percentage of apoptosis increase in H1299 cells with vector, SEMG1 or SEMG2 overexpressing after treatment with etoposide or cisplatin. Results are represented as mean ± SEM of three experiments. **P < 0.01.

To address this, we first we carried out an MTT assay using our H1299 NSCLC model cell lines with SEMG1or SEMG2 overexpression. Cells were treated with different concentrations of doxorubicin and cisplatin. As shown on Fig. 5a, b, both SEMGs sensitized H1299 cells to both genotoxic drugs. At the same time, H1299 cells with knockdown of SEMG2 (note, that SEMG1 is not expressed in these cells) were slightly more resistant to the treatment with these drugs (Fig. 5c). Taken together, these results suggest that SEMGs affects the susceptibility of malignant cells to genotoxic drugs.

Next, H1299 cells overexpressing either SEMG1 or SEMG2 were treated for 24 h with 75 μM of etoposide or 30 μM of cisplatin followed by apoptosis analysis using Annexin V staining. As shown in Fig. 5d, increased expression of either SEMGs (especially SEMG1) on its own, without any additional treatment, increased the population of Annexin V-positive cells to 70% (SEMG1) and 20% (SEMG2), respectively. This means that forced expression of SEMGs primed these cells to apoptosis. In accord with the data obtained, the treatment of these cells with 75 uM etoposide increased the number of Annexin V-positive cells to 8% (in the case of SEMG2) and 24% (in the case of SEMG2), respectively. Further, treatment of cells with another DNA damaging agent, cisplatin, enhanced the population of Annexin V-positive cells to 27% (for SEMG1) and 14% (for SEMG2), respectively (Fig. 5e).

Taken together, these data suggest that SEMGs enhance the level of apoptosis and sensitize cancer cells to genotoxic drugs.

## DISCUSSION

Rodrigues with colleagues^24^ has shown that SEMGs occur in SCLC and only in minority of NSCLC cell lines. However, we demonstrate here the ubiquitous presence of SEMG2 in all NSCLC cell lines tested whereas SEMG1 was detected in three out of five cell lines: in H520 squamous carcinoma and in two adenocarcinoma (H522 and H1650) cell lines. Thus, these data suggest that SEMGs are frequently expressed not only in SCLC, but also in NSCLC and other solid tumors (O.S. and N.B. unpublished observations). Berti with collegues^25^ detected SEMGs in the serum of NSCLC patients. These results support our findings.

Using immunofluorescence microscopy, we have shown that SEMGs have cytoplasmic localization in H520 cells. In contrast, SEMGs are predominantly nuclear in H1299 and H1650 cells. It would be interesting to see whether these dot-like nuclear structures that SEMGs form in the nucleus overlap with PML bodies or other known nuclear structures^27^. Apparently, the sub-cellular localization of SEMGs depends on the cellular context and may reflect different biological functions of these closely related proteins.

It was shown previously^18^ that SEMGs possess ROS-scavenging activity which is critical for sperm fertility. Nevertheless, at least in our adenocarcinoma cancer cell model, we have shown that overexpression of both SEMG1 and SEMG2 elevated ROS production two-fold. It seems that ROS-scavenging activity or other biological properties of SEMGs strongly depends on the context of cells.

Strong up-regulation of the oxidative stress level can lead to various consequences to cells. We observed that both ectopically expressed SEMGs significantly impeded the proliferation of lung adenocarcinoma cells. Moreover, overexpression of SEMGs (SEMG1, in particular) augmented dramatically the number of Annexin V-positive cells, suggesting that these cells undergo apoptosis.

In addition, we have also shown that SEMG1 and SEMG1 sensitized H1299 cells to genotoxic drugs doxorubicin, cisplatin, and etoposide, which differ in the mechanism of DNA damage, but are similar in their ability to produce ROS.

Collectively, our data suggest tumor suppressive properties of SEMG1 and SEMG2 in model H1299 lung adenocarcinoma cells. Indeed, SEMGs belong to the class of CTAs that are frequently associated with aggressive, high grade tumors^2,10^. However, it should be noted that examples of positive association between CTAs expression and survival of patients are also reported^28,29^. In line with this, positive association between expression of SEMG2 and survival rates of patients in the case of prostate cancer was demonstrated^30^. Furthermore, a positive correlation between survival of patients and high levels of SEMG1 expression has been observed in case of renal carcinoma^22^.

Taken together, SEMGs arguably play a positive role in tumorigenesis by sensitizing NSCLC cells to genotoxic therapy. This finding is especially peculiar given the published negative role of SEMGs in neoplasia. These diametrically opposite effects of SEMGs on cancer cells can be explained by differences in their origin. Irrespectively, this report has its value since it describes the first attempt to uncover effects of SEMGs on lung cancer cells at the molecular level. Additional studies are required to elucidate the biological role of SEMG1/2 in tumorigenesis.
